# Use of evidence-based approaches in procurement and implementation of health and welfare technologies – a survey among Swedish municipalities

**DOI:** 10.1186/s12913-023-10021-9

**Published:** 2023-09-22

**Authors:** Matt X. Richardson, Sarah Wamala Andersson

**Affiliations:** Department of Health and Welfare Technology, School of Health and Welfare, Mälardalen University, Eskilstuna, Sweden

**Keywords:** Health and welfare technology, Procurement, Implementation, Evidence, Municipal health and social care, Sweden

## Abstract

**Background:**

Health and welfare technologies (HWT) are increasingly procured and implemented by public providers in Swedish municipalities, but it remains unclear if and how evidence for these technologies’ effectiveness is used in both processes. The aim of this study was to investigate the use of evidence in Swedish municipal public sector procurement and implementation of HWT.

**Methods:**

A telephone survey of 197 municipalities was conducted with questions regarding the use of evidence in both processes, as well as eventual support needs regarding its use. Standard definitions of HWT and evidence were provided prior to the survey. Response frequencies and percentage proportions were calculated per question. Lambda (Λ) values with corresponding significance values were calculated for associations between responses to selected questions and the size and type of municipality, with values of 0.01 to 0.19 designated as weak associations, 0.20 to 0.39 as moderate, and 0.40 and above as strong.

**Results:**

Sixty-four municipalities completed the entire survey. Consistent use of evidence for effectiveness of HWT occurred in less than half of respondents’ municipal public procurement processes. Two-thirds of municipalities did not have an established model or process for implementation of HWT that used evidence in any manner. More than three quarters of municipalities lacked a systematic plan for follow-up and evaluation of effectiveness of implemented HWT, and of those that did less than half followed their plan consistently. Most municipalities expressed the need for support in using evidence in HWT-related processes but did not consider evidence and systematic evaluation to be prioritized.

**Conclusions:**

Weaknesses and gaps in using evidence in procurement and implementation processes may create a legacy of sub-optimal implementation of HWT in Swedish municipal health- and social care services, and lost opportunities for real-world evidence generation. There was a clear indication of the need for unified national guidance for using and generating evidence in key HWT-related municipal processes and implementation. Such guidance needs to be developed and effectively communicated.

**Supplementary Information:**

The online version contains supplementary material available at 10.1186/s12913-023-10021-9.

## Background

Health and welfare technologies (HWT) *are technology-based interventions that aim at maintaining or promoting health, wellbeing, quality of life and/or increasing efficiency in the operational delivery of welfare, social and health care services, while improving working conditions of the staff* [1, 2]. Examples of HWT include digital devices and applications for user safety and security, health monitoring and treatment, but also competency development and decision support for professionals. Increased self-management and care, and a reduced need of in-patient or institutionalized care, are typical goals associated with HWT use.

HWT are sometimes, but in many cases not, regulated according to the EU Medical Devices Regulation (MDR) [3]. This is largely dependent on a technology’s intended use claims, where many HWT uses fall outside of the MDR’s jurisdiction despite having potentially significant health, well-being, and/or efficiency outcomes. Examples of exempted HWT include various digital safety alarms, remote surveillance systems, and many health guidance and self-management applications. The requirements for evidence for effectiveness placed on such HWT outside of this regulation are therefore more difficult for end users, providers, developers and other stakeholders to define and/or assess [4]. Guidance on how to generate evidence is also more difficult to coordinate, particularly regarding the lifespan and multiple possible applications of a technology, possibly due to the heterogeneity of the fields of expertise required to develop such technologies [1].

Sweden’s publicly funded health care system consists of independent national, regional, and municipal levels, the latter of which are the primary purchasers of HWT [5, 6]. While the country’s 21 regions provide hospital and general practice-based care, its 290 municipalities provide post-hospital care, home care, and specialized home- and institutional care for elderly and those with functional disability or variance. The national level establishes overarching policies, guidelines, knowledge support, and supervision to the regions and municipalities through its agencies, as well as a national vision of increased HWT implementation and use in healthcare and social services [7]. In line with this vision, and a considerable amount of nationally earmarked funding [8–10], municipal procurement and implementation of HWT has increased dramatically. 72% of municipalities stated in 2021 that they have policy documents for implementation or use of e-Health or welfare technologies [6]. The Swedish Association of Local and Regional Authorities (SALAR) has also prioritized such implementation and use for a handful of HWT [11] including digital safety alarms (used in 100% of municipalities by approximately ~ 203 000 persons in 2021), Global Positioning System-based alarms (62% of municipalities, ~ 1 800 persons in 2021), and digital nocturnal surveillance (72% of municipalities, ~ 3 150 persons in 2021) [6].

Although other European countries have endorsed national strategies [12, 13], Sweden currently lacks unified national guidance for municipalities on how HWT-specific procurement, implementation and evaluation should take place. Currently, there are five national agencies and one member organisation with tasks that may relate to this:

1. The National Board of Health and Welfare (*Socialstyrelsen*) develops and provides knowledge support to health care and social services so they can be conducted from an evidence-based perspective [14].
2. The Swedish Agency for Health Technology Assessment and Assessment of Social Services (*Statens beredning för medicinsk och social utvärdering, SBU*) assesses health care and social services interventions mainly through systematic reviews and professional consultation [15].
3. The Agency for Digital Government (*Myndigheten för Digital Förvaltning, DIGG*) supports public sector digitalisation with a focus on effectiveness through standards, formats, specifications, and data transfer requirements [16].
4. The National Agency for Public Procurement (*Upphandlingsmyndigheten*) supports sustainability and competency in public sector procurement of innovative products and services, among others. They co-operate with DIGG and SALAR in this work [17].
5. The National e-Health agency (*E-hälsomyndigheten*) coordinates the national government’s e-health initiatives and analyses developments in the field of e-health [18].
6. SALAR (*Sveriges kommuner och regioner*) is the member organisation for all Swedish regions and municipalities, and it supports digitalisation in health and social care services, including operating a national government-funded welfare technology competency centre [8].

Recent systematic reviews show a lack of high-quality evidence for many expected effects of HWT that are broadly implemented in Sweden [19, 20], and that expected or acceptable levels of value are not reached [21]. Another recent systematic review of procurement practices in Sweden showed that requirements for, and assessment of, evidence for technologies’ effectiveness is rarely used during the procurement process. This is in contrast to the national guidance regarding evidence-based decision-making processes for care, the formulation of which is the responsibility of the National Board of Health and Welfare and the SBU [22]. It has not been established if the presence or absence of evidence requirements in the procurement process, which begins “upstream” to implementation, affects generation and evaluation of evidence during HWT employment and use. The National Agency for Public Procurement and SALAR, both of which support this process, do not appear to address evidence in explicit terms in their available guidance, however.

In this study we investigated the use of evidence of health and welfare technologies’ effectiveness in Swedish municipal public sector procurement and implementation processes, to answer the following primary questions:

- What type of evidence for HWT effectiveness during procurement is required by municipalities, if any?
- How do municipalities use evidence for HWT effectiveness during implementation?
- How do municipalities follow-up and evaluate HWT effectiveness after implementation?
- What kind of support, if any, do municipalities desire regarding use of evidence in their procurement, implementation, and evaluation processes?

## Methods

Following an initial pilot and validation study [23] of five Swedish municipalities in collaboration with SALAR, we employed an analysis- and investigation company (Novus Group International AB, Stockholm, Sweden) to conduct a telephone survey of all remaining municipalities in Sweden. Publicly available contact information for municipalities was used to send an initial query to identify and obtain the contact details of the appropriate organisational unit and/or individual(s) that could best respond to the survey topics. Follow-up communication was conducted as necessary to identify the most appropriate respondents. These were then used to finalize the list of individuals to be recruited to the study. Recruited respondents were given the opportunity to book a time that best suited their schedule for the telephone survey. Municipal officials that agreed to participate in the study signed informed consent forms regarding their participation as representatives of their respective municipalities’ activities. All queries and responses were therefore available as public documents according to Swedish public right of access to information regulations. As participants were responding as official representatives the study was exempted from ethical approval application following internal review involving the research team, departmental research coordinators, and the departmental head at Mälardalen University. Participation was, however, voluntary and in accordance with the Declaration of Helsinki. All information that could be used to identify respondents and/or their respective municipalities were anonymised by investigation company prior to the delivery of the data set to the research team.

The telephone survey was conducted between February 10–27, 2022. Interviews took approximately 20 min on average to conduct. The questions formulated by the researchers and posed in the survey can be found in Supplement 1.

To create a common understanding, key definitions were provided prior to the survey and interviews, including for *health and welfare technology* (same as in background section, [2]) and *evidence for effectiveness* (“a basis that supports the perception that a technology leads to the desired or expected effect”).

### Analysis

Responses were compiled and analysed descriptively with response frequencies and percentage proportions calculated. Associations between size and type of municipality and responses in some of the survey questions were described based on the Lambda (Λ) coefficient [24] using SPSS with corresponding approximate significance values, after cross-tabulation analyses. For Lambda statistics, values of 0.01 to 0.19 were designated as weak associations, 0.20 to 0.39 as moderate, and 0.40 and above as strong associations.

## Results

A summary of the main results, including the recruitment and respondent population size, are presented in Table 1. Question-specific response rates are presented in the remainder of the results.

**Table 1:** Summary of main results

Variable	Value
A. Number of municipalities that provided a relevant organization/person to contact for response	197
B. Percentage of A. that completed the interview	32% (n = 64)
C. Percentage of B. that had procured HWT in the previous 12 months	78% (n = 50)
D. Percentage of C. that always or often required evidence for HWT effectiveness.	44% (n = 33)
E. Percentage of B. that had implemented HWT in the previous 12 months	84% (n = 54)
F. Percentage of E. that had an established model for HWT implementation	15% (n = 8)
G. Percentage of E. that systematically followed-up and/or evaluated implemented HWT	28% (n = 15)
H. Percentage of B. that desired support in using evidence during procurement, implementation, follow-up and/or evaluation of HWT	63% (n = 40)

### Respondents

The sizes and types of municipalities in the study population can be seen in Table 2, for both respondents and non- or incomplete respondents; the latter includes 17 interviews that were initiated but could not be completed entirely. The respondent municipalities were located in 61% of the Swedish regions with an even geographical distribution throughout the country.

**Table 2:** Respondent and non-respondent municipalities by type, N (percentage of total contacted municipalities)

Municipality type^1^	Respondents	Non- or incomplete respondents
Commuting municipality near large city	14 (7.1%)	14 (7.1%)
Commuting municipality near medium-sized town	12 (6.1%)	25 (12.7%)
Rural municipality	11 (5.6%)	29 (14.7%)
Commuting municipality near small town	9 (4.6%)	26 (13.2%)
Small town	8 (4.1%)	11 (5.6%)
Rural municipality with tourism industry	4 (2.0%)	7 (3.6%)
Low commuting municipality near medium-sized town	3 (1.5%)	11 (5.6%)
Medium-sized town	2 (1.0%)	9 (4.6%)
Large city	1 (0.5%)	1 (0.5%)
**Total**	**64 (32.5%)**	**133 (67.5%)**

^1^Large cities: < 200 000 inhabitants; medium-sized towns: < 50 000 ≥ 200 000 inhabitants; small towns < 15 000 ≥ 40 000 inhabitants; rural municipalities > 15 000 inhabitants [25]

Of the representative persons for the respondent municipalities, 33% were managers responsible for the municipalities’ health or social care services, or a unit within those services. 53% were digitalisation or IT managers, strategists or similar, and a further 11% were development managers or similar. 3% of respondents had a different title or function than those above.

### Procurement

**Fig. 1:**
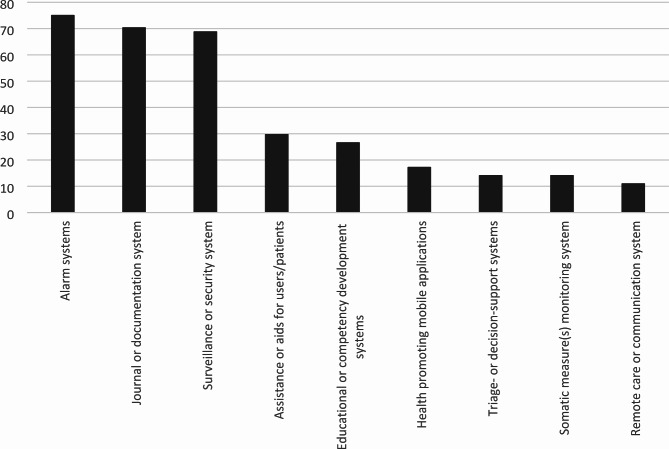
Type(s) of HWT procured by municipalities in the last 12 months, percent values (n = 50)

**Fig. 2:**
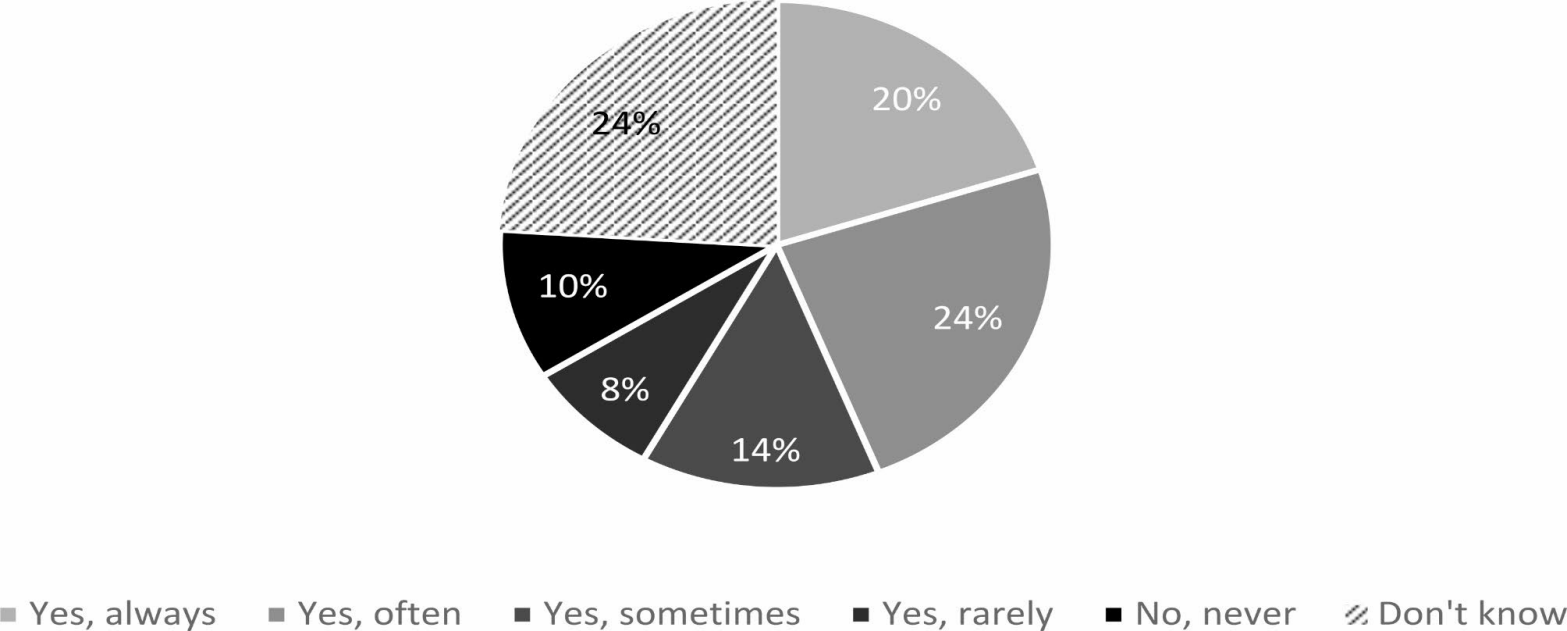
Extent of requirement of evidence for effectiveness by municipalities when procuring HWT in the last 12 months (n = 50)

The types of HWT that had been procured by 78% of the respondent municipalities in the past 12 months can be seen in Fig. 1. 11% had not procured any HWT during the same period, and the remaining 11% did not know if procurement had taken place. A majority of procuring municipalities only occasionally or never required evidence for HWT effectiveness during the procurement process (Fig. 2). There was a weak association between municipality type and procurement in the last 12 months (Λ = 0.14, p = 0.31), and a moderate association with requiring evidence for HWT effectiveness (Λ = 0.27, p = 0.12), with large and medium-sized municipalities and nearby commuting centers more likely to have procured and require evidence.

**Fig. 3:**
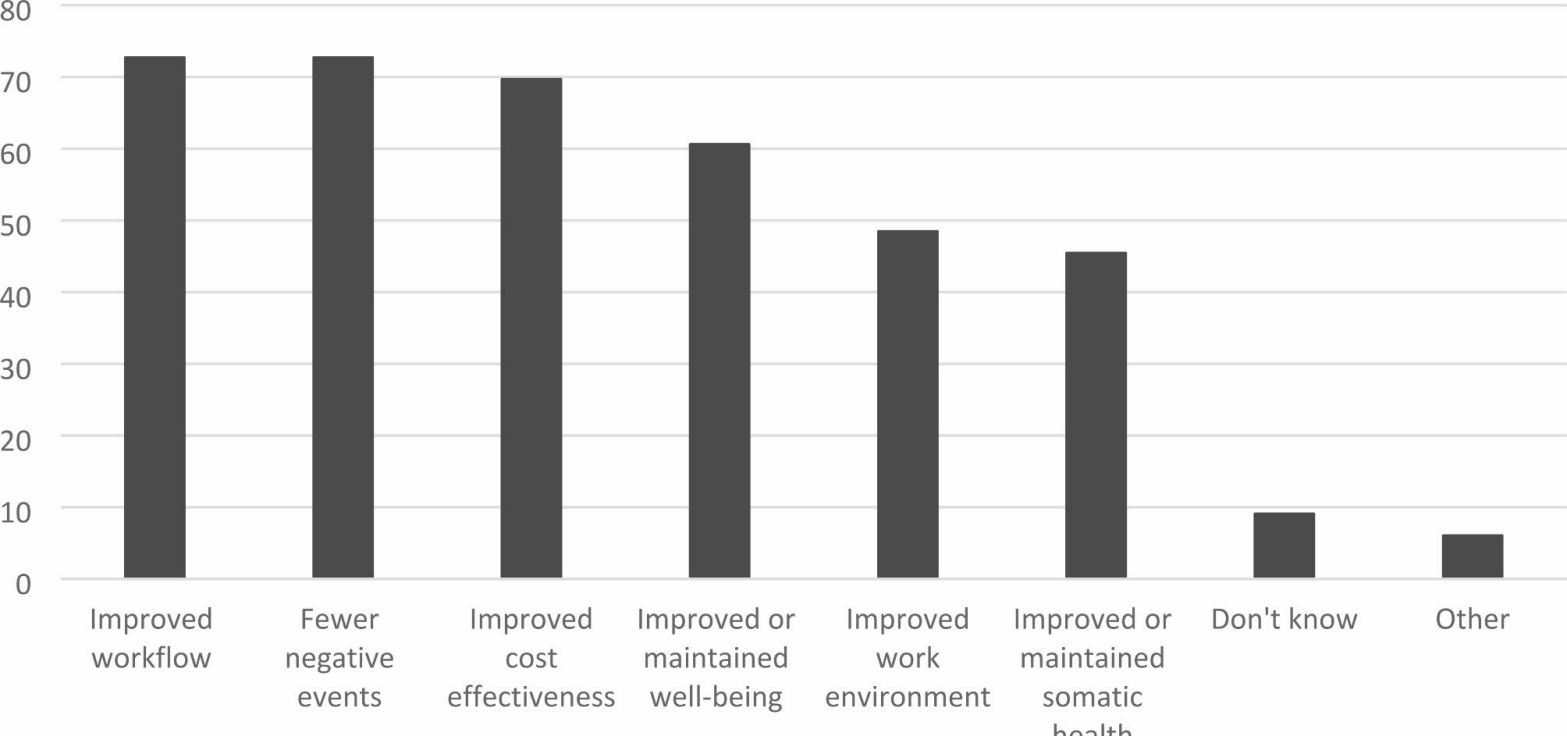
Outcomes for which municipalities required evidence when procuring HWT, percent values (n = 33)

**Fig. 4:**
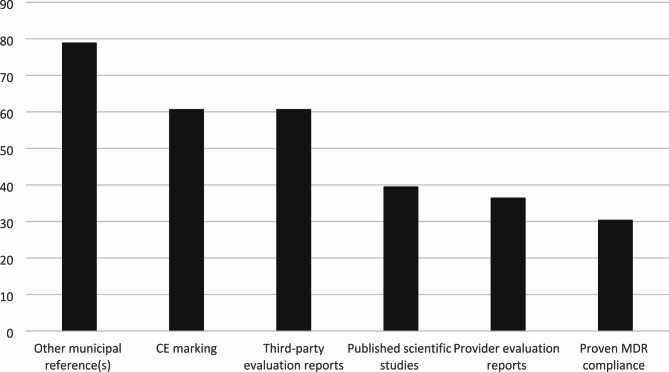
Sources of evidence requested and/or accepted by municipalities when procuring HWT, percent values (n = 33)

For those municipalities that to any extent required evidence for HWT effectiveness (N = 33), most required evidence for more than one outcome (Fig. 3) and from more than one source or format (Fig. 4).

In 85% of municipalities, persons within the organisation assessed the evidence that was gathered, and in 76% of cases this was resources responsible for the procurement at hand. 61% of municipalities involved other internal expertise regarding the area or technology in focus for procurement, while 21% used persons outside of their own organisation to assist in assessment of evidence. 6% did not know which resources were responsible for evidence assessment during procurement.

The evidence obtained during the procurement process for those municipalities that required it was used to assign points or rank the submitted bids in 52% of municipalities, and in 48% to determine if bids were qualified. 15% of municipalities used evidence obtained to make recommendations to the organisational unit that initiated the request for tender, while 6% used it for other purposes. 21% did not know how the evidence was used in the remainder of the procurement process.

### Implementation

**Fig. 5:**
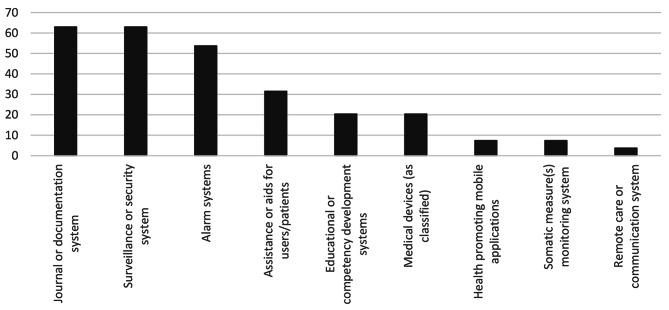
Types of HWT implemented by municipalities in the last 12 months, percent values (n = 54)

The types of HWT that had been implemented by 84% of respondent municipalities in the past 12 months is presented in Fig. 5. 9% had not implemented any HWT during the same period, and the remaining 6% did not know if implementation had taken place. There was no association between municipality type and implementation history.

76% of municipalities that had implemented or begun implementing HWT in the past twelve months lacked a process or model for such implementation, and 9% did not know. Similarly, 66% of the same municipalities lacked a plan for systematic follow-up and evaluation of the implemented HWT’s effectiveness, and 11% did not know. There was no association between municipality type and presence of implementation process or model or systematic follow-up and evaluation plan.

**Fig. 6:**
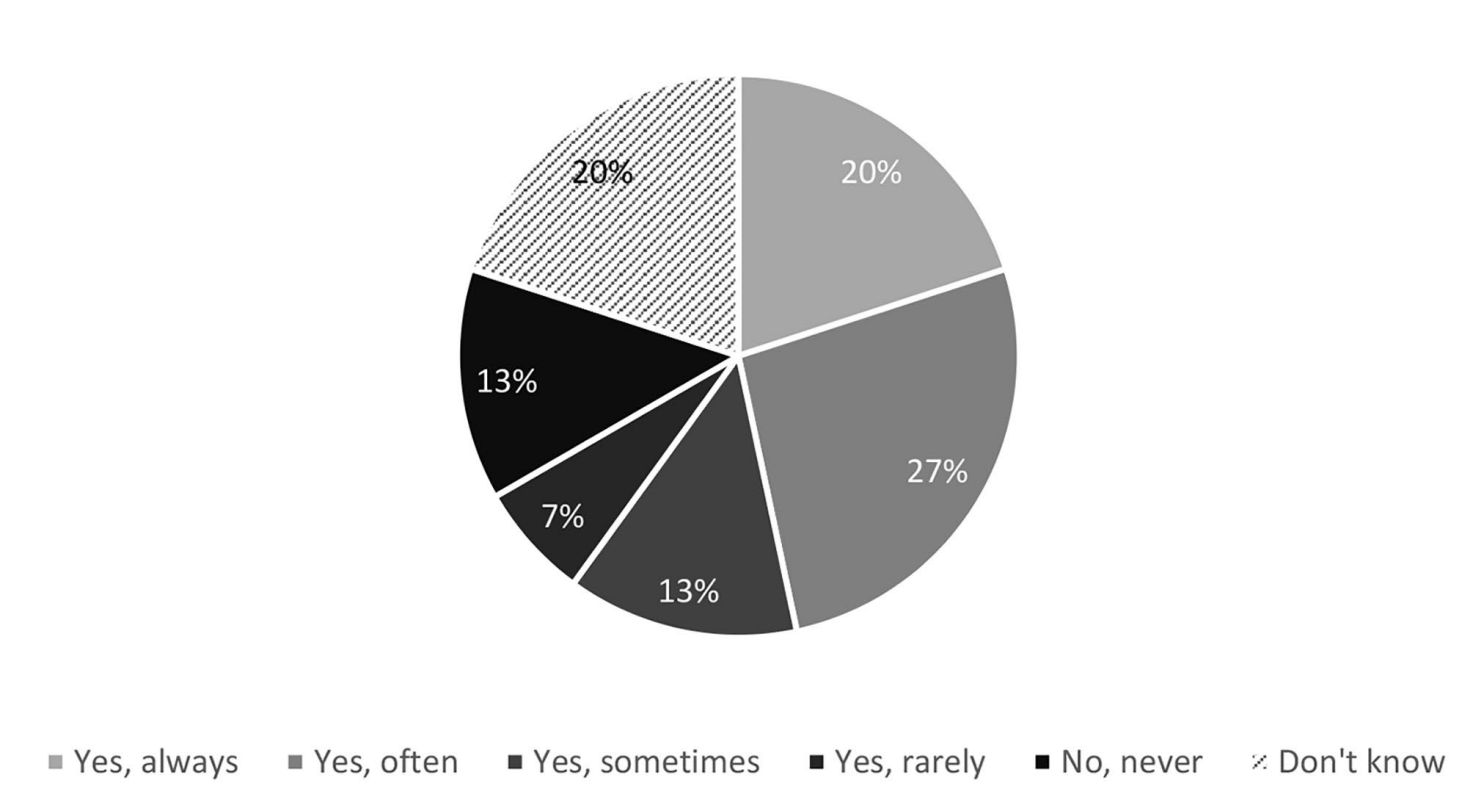
Percentage of municipalities (n = 15) that follow their established plan for follow-up and/or evaluation of HWT

Of those municipalities that did have a plan for systematic follow-up and/or evaluation of HWT effectiveness (n = 15), 47% followed the plan always or most of the time (Fig. 6). The follow-up and/or evaluation were most often conducted by personnel who used the HWT in question (87%), followed by other employees in the organisation (67%), the provider of the technology (33%), or by independent third parties including researchers (27%). 13% did not know what resources conducted the follow-up and/or evaluation.

**Fig. 7:**
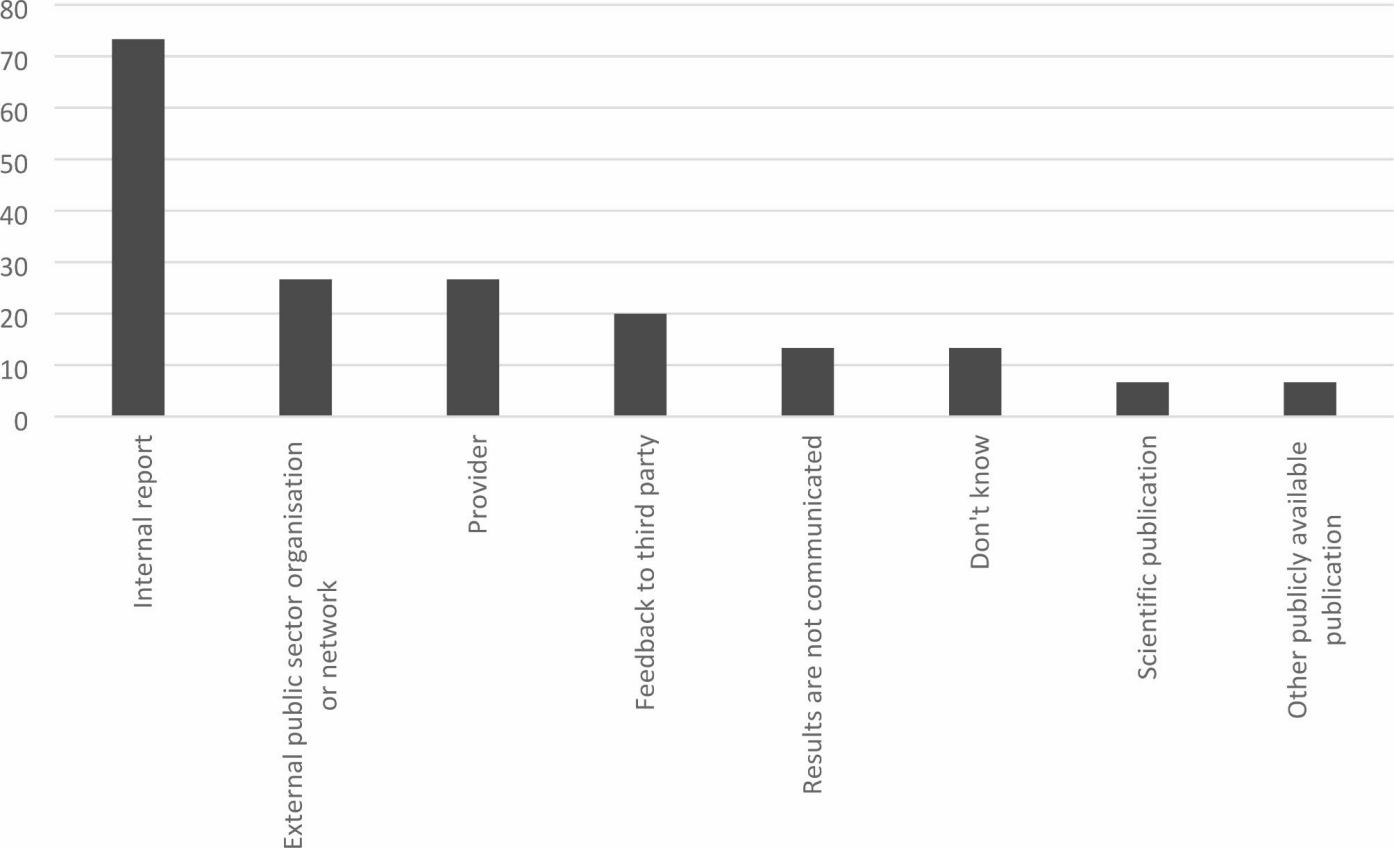
Channels used by municipalities to disseminate follow-up and/or evaluation findings regarding HWT effectiveness, percentage values (n = 15)

Of the municipalities that conducted follow-up and/or evaluation of HWT effectiveness (n = 15), a majority disseminated the results of this via internal report, while others used other forms of publication or communication through networks and external organisations (Fig. 7). 13% did not know how results were disseminated, and 13% did not disseminate results in any structured manner. The results of the follow-up and/or evaluation was used to adjust the HWT in operation always or most of the time in 33% of municipalities, while 53% stated that it was used for such purposes sometimes or rarely and 13% did not know.

### Need for support in evidence-based work

Of the respondent municipalities, 33% felt that evidence and systematic follow-up and evaluation of this was prioritized in their organisation, while 48% did not and 19% did not know. 63% desired some form of support in using evidence when procuring and implementing HWT in their organisation, while 16% did not and 22% did not know. There was a moderate association between municipality type and prioritization of evidence, follow-up, and evaluation (Λ = 0.24, p = 0.27), with larger municipalities prioritizing more. There was no association between municipality type and desire for support.

From those municipalities desiring support (n = 40), several open field responses were collected. A majority of these could be categorized into the following themes:

- expertise support, e.g., area-specific knowledge banks or capabilities regarding methods or results interpretation (28%);
- process guidance support, e.g., templates, models, or workflows that could be followed for evidence-related procurement tasks (42%);
- resource support, e.g., personnel to assist with evidence generation tasks during implementation (12%); and.
- organisational support, e.g., increased partnership with other organisations via networks, forums or to conduct common tasks related to evidence (9%).

## Discussion

A minority of Swedish municipalities that purchase and implement HWT for health and social services report that they consistently use evidence in these processes. They also report that they desire increased support in how to improve the use of evidence for, and systematic evaluation of, HWT effectiveness, but that it is currently of low priority. This appears to be true regardless of municipality type, although there is some indication that larger municipalities at least feel that the use of evidence and systematic evaluation is more prioritized.

As far as we know, this the first large-scale survey addressing the use of evidence in procurement and implementation process of HWT in Swedish municipalities.

The limited use of evidence in procurement reported by municipalities aligns with the findings of a recent systematic review of Swedish requests for tenders when procuring HWT [5], although it is higher (44%) than what was identified in the review, where less than a fifth of procurements contained criteria requiring evidence for effectiveness. The explanations for this difference are uncertain. It is possible that the use of evidence is overreported in the current study, or that improvements in its use have increased since the period for which the review was conducted (prior to May 2021).

The lack of implementation and evaluation models and processes for HWT presents a significant obstacle for municipalities in using or systematically generating evidence for effectiveness. The lack of unified national guidance for municipalities on how implementation and evaluation of HWT should take place is a potential contributor to this obstacle. Despite guidance and support tasks among four national agencies and one member organisation, the responses in this survey appear to indicate that these organisations’ support is inadequate, unused, and/or not well known to municipalities. This may lead to considerable differences in both operations and outcomes among municipal authorities delivering health and social care services. In turn these may contribute to variations and inequalities in access to technologies among end-users. The evidence-based guidelines that national agencies produce have been described as a form of “recentralization” of decision-making power within Sweden’s devolved health care system of autonomous regional and municipal care providers [26]. The current results suggest, however, that such an effect is not forthcoming regarding HWT use. A more nationalized, central steering of HWT implementation is currently being planned by the government [27], although its eventual effects on evidence use are not explicitly mentioned.

The lack of consistent use of evidence for HWT effectiveness during procurement processes may initiate a “cascade of ambiguity” regarding digital technologies’ benefits and cost-effectiveness among health and social care organisations, their users and society at large. The absence of processes for implementation and subsequent systematic evaluation of HWT effectiveness reported by most municipalities may further increase the magnitude of this ambiguity, and further reduce cost-effectiveness. The resultant organizational failure to use evidence during critical phases of HWT purchasing, introduction and employment presents a clear and significant risk for creating a legacy of not only sub-optimal technologies in health and social care services, but also potentially ineffective or harmful ones.

## Conclusions

There is a low level of evidence use among Swedish municipalities when procuring and implementing HWT, yet a high demand for support in how to use it. Unified national guidance for HWT-specific, evidence-based procurement and implementation at the municipal level is recommended to increase the employment of effective digital technologies in health and social care services. This may especially benefit smaller municipalities that may lack the partnerships or capacity to conduct such processes on their own, as well as during unique or novel procurements in any municipality. Further research is needed to address how such a national guidance can be designed and optimally communicated to ensure that it supports municipalities and ultimately contributes to equitable access of technologies to end-users. Research on long-term outcomes based on other countries´ experiences that have adapted national strategies and guidelines for digital health technologies is also needed.

### Strengths and limitations

Using an interview-based survey in this study may have reduced potential response bias related to misunderstanding of survey questions. The respondents represented relevant stakeholders that managed the procurement and implementation process of HWT in municipalities.

Although the intention of the current study was to collect data in all municipalities, the final response and completion rate was relatively low, which may limit the applicability of the results to all Swedish municipalities. However, it should be noted that, the obtained responses were representative of all municipality types and geographical locations across the country. It is worth noting that differences in democratic and governance structures in the Swedish health system may limit the applicability of the results to other countries with different systems.

The study adheres to the STROBE guidelines for observational cross-sectional studies.

### Electronic supplementary material

Below is the link to the electronic supplementary material.

**Supplement 1**. Survey questions (English translation).

## Data Availability

The datasets used and/or analysed during the current study are available from the corresponding author on reasonable request.

## List of abbreviations

HWT: Health and welfare technologies
MDR: Medical Devices Regulation
SALAR: Swedish Association of Local and Regional Authorities

## References

[1] Andersson SW, Richardson MX, Cozza M, Lindén M, Redekop K (2021). Addressing evidence in health and welfare technology interventions from different perspectives. Health Policy and Technology.

[2] Wamala-Andersson S, Richardson MX, Cozza M, Lindén M, Redekop K (2021). Addressing evidence in health and welfare technology interventions from different perspectives. Health Policy and Technology.

[3] Medical Devices Regulation. EU 2017/745 (2017).

[4] Guo C, Ashrafian H, Ghafur S, Fontana G, Gardner C, Prime M. Challenges for the evaluation of digital health solutions—A call for innovative evidence generation approaches. npj Digit Med. 2020;3(1). 10.1038/s41746-020-00314-2 PMC7453198 32904379

[5] Richardson MX, Landerdahl Stridsberg S, Wamala Andersson S (2022). Evidence-related requirements in swedish public sector procurement of health and welfare technologies – a systematic review. BMC Health Serv Res.

[6] National Board of Health and Welfare (Socialstyrelsen). E-Health and welfare technology in the municipalities 2021 (E-hälsa och välfärdsteknik i kommunerna 2021). (Swedish). Socialstyrelsen. 2021. Available from: https://www.socialstyrelsen.se/globalassets/sharepoint-dokument/artikelkatalog/ovrigt/2021-5-7384.pdf. Accessed 19 October 2022.

[7] Government Offices of Sweden (Regeringskansliet). Vision e-health 2025 (Vision e-hälsa 2025). (Swedish). Ministry of Health and Social Affairs, Ministry of Enterprise and Innovation. 2016. Available from: https://ehalsa2025.se/wp-content/uploads/2021/02/vision-e-halsa-2025-overenskommelse.pdf. Accessed 19 October 2022.

[8] Government of Sweden (Regeringen). Approval of an agreement between the state and the Swedish Association of Local and Regional Authorities regarding elderly care – technology, quality, and effectiveness with the elderly in focus. (Godkännande av en överenskommelse mellan staten och Sveriges Kommuner och Regioner om äldreomsorg – teknik, kvalitet och effektivitet med den äldre i fokus). (Swedish). Ministry of Health and Social Affairs. 2020. Available from: https://skr.se/download/18.4829a209177db4e31aa34433/1615454129940/2020_overenskommelse_aldreomsorg_teknik_kval_effektivitet.pdf. Accessed 19 October 2022.

[9] Government Offices of Sweden (Regeringskansliet). Agreement on support for an evidence-based practice for good quality in social care. (Överenskommelse om stöd till en evidensbaserad praktik för god kvalitet inom socialtjänsten.) (Swedish). Ministry of Health and Social Affairs. 2012. Available from: https://www.regeringen.se/overenskommelser-och-avtal/2013/01/s20128764fst/. Accessed 19 October 2022.

[10] National Board of Health and Welfare (Socialstyrelsen). Reporting of 2018 state transfers to municipalities for investments in welfare technology in care services. (Redovisning av 2018 års statsbidrag till kommuner för investeringar i välfärdsteknik inom omsorgen.) (Swedish). 2019. Available from: https://statsbidrag.socialstyrelsen.se/globalassets/dokument/instruktioner/statsbidrag-investeringar-valfardsteknik-inom-omsorgen-2019-rapport.pdf. Accessed 19 October 2022.

[11] Swedish Association of Local and Regional Authorities (SKR). Support and guidance for welfare technologies in elderly care 2021. (Stöd och vägledning välfärdsteknik äldreomsorg 2021.) (Swedish). 2021. Available from: https://skr.se/skr/integrationsocialomsorg/socialomsorg/aldre/overenskommelsealdreomsorg/stodochvagledningvalfardsteknik.52850.html. Accessed 19 October 2022.

[12] Digital Health Applications Ordinance (Digitale Gesundheitsanwendungen-Verordnung - DiGAV). (German Federal Institute for Drugs and Medical Devices, German.). 2020.

[13] NICE. Evidence Standards Framework for Digital Health Technologies. Update August 2022. National Institute for Health and Clinical Excellence (NICE); 2018.

[14] Proclamation and instruction for the Swedish National Board of Health. and Welfare (Förordning med instruktion för Socialstyrelsen), Stat. SFS 2015:284 (2015). (Swedish). Available from: https://www.riksdagen.se/sv/dokument-lagar/dokument/svensk-forfattningssamling/forordning-2015284-med-instruktion-for_sfs-2015-284. Accessed 19 October 2022.

[15] Proclamation and instruction for the Swedish Agency for Health Technology Assessment and Assessment of Social Services. (Förordning med instruktion för Statens beredning för medicinsk och social utvärdering), Stat. SFS 2007:1233 (2008-01-01, 2007). (Swedish). Available from: https://www.riksdagen.se/sv/dokument-lagar/dokument/svensk-forfattningssamling/forordning-20071233-med-instruktion-for_sfs-2007-1233. Accessed 19 October 2022.

[16] Proclamation and instruction for the Agency for Digital Government. (Förordning med instruktion för Myndigheten för digital förvaltning), Stat. SFS 2018:1486 (2018-07-05, 2018). (Swedish). Available from: https://www.riksdagen.se/sv/dokument-lagar/dokument/svensk-forfattningssamling/forordning-20181486-med-instruktion-for_sfs-2018-1486. Accessed 19 October 2022.

[17] Proclamation and instruction for the National Agency for Public Procurement. (Förordning med instruktion för Upphandlingsmyndigheten), Stat. SFS 2015:527 (2015-07-23, 2015). (Swedish). Available from: https://www.riksdagen.se/sv/dokument-lagar/dokument/svensk-forfattningssamling/forordning-2015527-med-instruktion-for_sfs-2015-527. Accessed 19 October 2022.

[18] Proclamation for the National E-health Agency. (Förordning med instruktion för E-hälsomyndigheten), Stat. SFS 2013:1031 (2013-12-05, 2013). (Swedish). Available from: https://www.riksdagen.se/sv/dokument-lagar/dokument/svensk-forfattningssamling/forordning-20131031-med-instruktion-for_sfs-2013-1031. Accessed 19 October 2022.

[19] Ehn M, Richardson MX, Stridsberg SL, Redekop WK, Wamala-Andersson S. Mobile safety alarms based on global positioning system technology in care of older adults: a systematic review of evidence based on a general evidence framework for digital health technologies. J Med Internet Res. 2021. 10.2196/27267 PMC8546532 34633291

[20] Richardson MX, Ehn M, Stridsberg SL, Redekop K, Wamala-Andersson S (2021). Nocturnal digital surveillance in aged populations and its effects on health, welfare and social care provision: a systematic review. BMC Health Serv Res.

[21] Frennert S, Baudin K. The concept of welfare technology in Swedish municipal eldercare. Disabil Rehabil. 2019:1–8. 10.1080/09638288.2019.1661035 31503509

[22] Official reports of the Swedish Government (Statens offentliga utredningar). An evidence-based practice in social care that benefits the user (En evidensbaserad praktik inom socialtjänsten till nytta för brukaren). (Swedish). SOU 2008:18. Stockholm: Fritzes. 2008. Available at: https://www.regeringen.se/49b6a8/contentassets/c33ab37b7bcc4512a1d07992ddfad267/evidensbaserad-praktik-inom-socialtjansten---till-nytta-for-brukaren-sou-200818. Accessed 19 October 2022.

[23] Norgren T, Richardson MX, Wamala-Andersson S (2023). Obstacles to evidence-based procurement, implementation, and evaluation of Health and Welfare Technologies in Swedish Municipalities: mixed methods study. JMIR Form Res.

[24] Goodman L, Kruskal W (1954). Measures of association for cross classifications. J Am Stat Assoc.

[25] Swedish Association of Local and Regional Authorities (SKR). Classification of Swedish municipalities 2017. 2016. Available from: https://skr.se/download/18.4d3d64e3177db55b16631b96/1615474478946/Classification%20of%20Swedish%20Municipalities%202017.pdf. Accessed 19 October 2022.

[26] Fredriksson M, Blomqvist P, Winblad U. Recentralizing healthcare through evidence-based guidelines - striving for national equity in Sweden. 2014;14(509). 10.1186/s12913-014-0509-1 PMC4226849 25370710

[27] Government Offices of Sweden (Regeringskansliet). Assignment to create a proposed roadmap for implementation of a national digital infrastructure for health and medical care. (Uppdrag att ta fram ett förslag till färdplan för genomförandet av en nationell digital infrastruktur för hälso- och sjukvården.) (Swedish). Ministry of Health and Social Affairs. 2023. Available from: https://www.regeringen.se/regeringsuppdrag/2023/06/uppdrag-att-ta-fram-ett-forslag-till-fardplan-for-genomforandet-av-en-nationell-digital-infrastruktur-for-halso--och-sjukvarden/. Accessed 19 August 2023.

