# Supplementary material for: Use of evidence-based approaches in procurement and implementation of health and welfare technologies – a survey among Swedish municipalities
Source: BMC Health Serv Res. 2023 Sep 22;23:1024. doi: 10.1186/s12913-023-10021-9 (PMC10517470; doi:10.1186/s12913-023-10021-9)
Supplement: Supplementary file 1 — Supplement 1. Survey questions (English translation). [file 12913_2023_10021_MOESM1_ESM.docx]

# Supplement 1. Survey questions (English translation).

Section 1: Respondent details

1. My name is:

2. My work title/position is:

Section 2: Procurement of HWT

*The following statements are about procurement of HWT in your organisation. Choose the answer from the alternatives that best describes your organisation in relation to the statement (in some cases you can choose more than one alternative). When choosing, assume that the procurement of HWT is announced publicly and that offers are submitted and judged. Assume as well that the procurement should be of ”normal” type for your organisation regarding cost and technical requirement(s) or function(s).*

3. My organisation has procured or ordered HWT via procurement during the last 12 months.

Yes

No (continue to question 10)

Don’t know (continue to question 10)

4. My organisation procures the following type(s) of HWT:

*Choose all alternatives that apply. If "Other" is chosen please provide a short description of the technology.*

Journal- or documentation system

Triage- or decision support system

Monitoring system for somatic measurements e.g. heart rate, blood pressure

Supervision or security system e.g. cameras or sensors for doors, beds, rooms

Assistive technologies for users/patients e.g. medication reminders, hygiene- or food preparation assistance

Distance- or remote communication systems

Alarm systems e.g. safety alarms, GPS, alarms

Health-promoting applications for mobile devices

Educational or competency development systems for personnel

5. My organisation requires evidence for the technology’s effectiveness when procuring HWT.

*Remember: Evidence for effectiveness is any basis that supports the perception that a technology leads to the desired or expected effect.*

Yes, often

Yes, sometimes

Yes, rarely

No, never (continue to question 10)

Don’t know (continue to question 10)

6. When procuring, my organisation requires evidence for the technology’s effectiveness regarding the following outcomes:

*More than one alternative can be chosen. If ”Other” is chosen please provide a short description of the outcome.*

Improved or maintained somatic health

Improved or maintained well-being

Fewer negative events or deviations

Improved cost effectiveness

Improved workflows or processes

Improved working conditions

Other

Don’t know

7.When procuring, my organisation accepts the following basis/bases as evidence for a technology’s effectiveness:

*More than one alternative can be chosen. If ”Other” is chosen please provide a short description of the basis.*

Published scientific studies

Evaluation or investigations conducted by consultants or third parties

Evaluation or investigations conducted by the HWT provider

Proven CE-certification

Proven adherence to the EU Medical Devices Regulation

Reference tenders from other purchases/procurers

Other

Don’t know

8. The following resources are used during procurement to assess the basis/bases that comprise evidence for a technology’s effectiveness:

*More than one alternative can be chosen. If ”Other” is chosen please provide a short description of the resource.*

One or more persons with expertise in the area

Employees within the organisation

Employees from external organisations

Responsible procurer/procurement group

We do not conduct assessments of the evidence basis/bases

Other

Don’t know

9. My organisation uses the following assessment methods during procurement for assessing the basis/bases that comprise the evidence for a technology’s effectiveness:

*More than one alternative can be chosen. If ”Other” is chosen please provide a short description of the method.*

Scoring/awarding points to assessed offers

Deciding if offers qualify in the procurement process

As a recommendation to the orderer or another part of the organisation

Other

Don’t know

Section 3: Implementation of HWT

*The following statements are about implementation of HWT in your organisation. Choose the answer from the alternatives that best describes your organisation in relation to the statement (in some cases you can choose more than one alternative). When choosing, assume implementation of HWT that has been procured or ordered via procurement by your organisation. Assume as well that the implementation is of ”normal” type for your organisation regarding cost and technical requirement(s) or function(s).*

10. My organisation has implemented, or started implementation, of HWT during the last 12 months.

Yes

No (continue to question 18)

Don’t know (continue to question 18)

11. My organisation has implemented, or started implementation of, the following type(s) of HWT during the last 12 months:

*More than one alternative can be chosen. If ”Other” is chosen please provide a short description of the technology.*

Medical devices (according to MDR)

Journal- or documentation system

Triage- or decision support system

Monitoring system for somatic measurements e.g. heart rate, blood pressure

Supervision or security system e.g. cameras or sensors for doors, beds, rooms

Assistive technologies for users/patients e.g. medication reminders, hygiene- or food preparation assistance

Distance- or remote communication systems

Alarm systems e.g. safety alarms, GPS, alarms

Health-promoting applications for mobile devices

Educational or competency development systems for personnel

12. My organisation has an established process or model that is used specifically for implementation of HWT.

Yes / No / Don’t know

13. My organisation has a plan for systematic follow-up and evaluation of HWT effectiveness.

Yes

No (continue to question 18)

Don’t know (continue to question 18)

14. My organisation follows up the specific requirements stated during procurement related to the technology’s effectiveness.

Yes, often

Yes, sometimes

Yes, rarely

No, never

Don’t know

15. My organisation uses the following approach(es) to follow-up the effectiveness of a technology during implementation:

*More than one alternative can be chosen. If ”Other” is chosen please provide a short description of the approach.*

Via personnel that use the technology

Via other employees within the organisation

Via third or independent parties

Via researchers / research studies

Via the provider of the technology

My organisation does not follow-up the technology’s effectiveness

Other

Don’t know

16. My organisation shares the result from follow-up and evaluation of the technology’s effectiveness in the following manner(s):

*More than one alternative can be chosen. If ”Other” is chosen please provide a short description of the manner(s).*

Via scientific publication

Via publication that is made available for the public

Via external network or organisation in the public sector

Via a report intended for internal use/feedback

Via feedback to or dialog with a third party

Via the provider

My organisation does not share the results of the follow-up of the technology’s effectiveness

Other

Don’t know

17. My organisation uses the results from follow-up and evaluation of implemented technology to make necessary adjustments to improve the technology’s effectiveness.

Yes, often

Yes, sometimes

Yes, rarely

No, never

Don’t know

18. In my organisation I feel that evidence for, and follow-up and evaluation of the technology’s effectiveness is prioritized.

Yes / No / Don’t know

Section 4: Support

19. According to you, does your organisation desire support, which you are not currently receiving, regarding how to procure, systematically follow-up and/or evaluate to be able to implement HWT with an evidence-based perspective?

Yes / No / Don’t know

20. If so, what type of support is desired?
